# Dr. Arthur Field Hinman

**Published:** 1915-03

**Authors:** 


**Dr. Arthur Field Hinman,** Syracuse, 1910; died in Clyde, N. Y., recently, of intestinal cancer, aged 31. Following his graduation, he served as interne in St. Joseph's Hospital of Syracuse and then engaged in practice in Auburn.

